# Dietary *Wolffia globosa* extract enhances innate immunity, serum biochemical responses, antioxidant defense, and expression of related gene in *Labeo rohita* under winter thermal stress

**DOI:** 10.3389/fimmu.2026.1864774

**Published:** 2026-06-18

**Authors:** Nitesh Kumar Yadav, Arun Bhai Patel, Parvind Kumar, Deepan Rajesh S., Himanshu Priyadarshi

**Affiliations:** 1 College of Fisheries, Central Agricultural University (Imphal), Agartala, Tripura, India; 2 College of Fisheries, Maharana Pratap University of Agriculture and Technology, Udaipur, Rajasthan, India

**Keywords:** antioxidant defense, immunomodulation, *Labeo rohita*, winter stress, *Wolffia globosa*

## Abstract

**Introduction:**

Temperature is a key environmental factor regulating physiological, biochemical, and immunological processes in ectothermic fish such as *Labeo rohita*. Exposure to low-temperature conditions during winter disrupts metabolism, suppresses immunity, and induces oxidative stress, thereby compromising fish health and productivity.

**Methods:**

*Labeo rohita* fingerlings (initial weight: 8.35 ± 0.50 g) were randomly distributed into four dietary treatments with three replicates each and fed isonitrogenous (25% crude protein) and isocaloric (16.5 MJ kg^−1^ gross energy) diets supplemented with graded levels of Wolffia extract (WGE; 0, 0.5, 1.5, and 3.0 g kg^−1^) for 75 days. The experiment was conducted in outdoor cemented tanks during the winter season (December–March) under natural low-temperature conditions (18–22 °C).

**Results:**

Dietary WGE significantly enhanced (P< 0.05) non-specific immune responses, with anti-protease activity increasing by 8.22–12.32%, myeloperoxidase by ~200–300%, respiratory burst by 76.92%, and lysozyme by up to 146.94% compared to the control. Carcass composition improved significantly, with crude protein reaching 18.01% and lipid 4.63%. Serum glucose decreased (15.78–49.26%), while total protein and albumin increased. Liver function markers, including SGOT, SGPT, ALP, and CK-MB, declined significantly, indicating improved hepatic integrity and reduced physiological stress. Antioxidant status improved, with elevated SOD and CAT activities and reduced MDA levels by up to 43.97% (liver) and 46.69% (serum). Gene expression analysis revealed downregulation of stress-related genes (*hsp70*: 0.042-fold; *hsp90*: 0.147-fold) and upregulation of immune-related genes (*c3*: 5.51-fold; *tnf-α*: 3.37-fold; *ifn-γ*: 7.73-fold).

**Conclusion:**

This study evaluated the protective effects of *Wolffia globosa* extract on innate immunity, antioxidant defense, and stress- and immune-related gene expression in *Labeo rohita*. Dietary WGE effectively mitigated cold-induced stress by improving immune function, antioxidant capacity, and metabolic performance. Among the tested levels, 1.5 g kg^−1^ WGE provided the most balanced physiological benefits, while higher inclusion levels induced stronger immune and antioxidant responses. These findings highlight the potential of *Wolffia globosa* as a sustainable functional feed additive for enhancing fish resilience under low-temperature conditions.

## 1 Introduction

Fish, being poikilothermic organisms, have body temperatures that fluctuate according to the surrounding environmental conditions, making temperature a critical factor regulating their survival, distribution, metabolism, and overall physiological performance (1). Because they lack an internal thermoregulatory mechanism, essential biological processes such as metabolism, growth, feeding activity, and immune competence are directly influenced by ambient water temperature (2). Consequently, deviations from the optimal thermal range can disturb metabolic homeostasis and physiological functions, while prolonged exposure to unfavorable temperatures may result in severe stress, impaired health, or even mortality (3).

Low temperatures during winter not only retard growth but also induce a cascade of physiological disturbances affecting metabolic regulation, oxidative balance, and immune function. Under such conditions, immune activity is markedly suppressed, making the maintenance of immunocompetence particularly challenging in teleosts (4). Both innate and adaptive immune components respond differentially to temperature fluctuations, with innate immune parameters such as phagocytic activity, respiratory burst, intracellular killing, and complement activity being especially sensitive to temperature decline (5). In parallel, cold stress disrupts mitochondrial function, leading to excessive production of reactive oxygen species (ROS) and subsequent oxidative stress, which compromises physiological performance and organismal fitness (6).

These challenges are particularly relevant in freshwater aquaculture, where seasonal temperature fluctuations are inevitable. In India, carp culture constitutes a major component of inland aquaculture, with rohu (*Labeo rohita*) being one of the most important species due to its high market demand, adaptability, and significant contribution to production (7). However, rohu is highly susceptible to seasonal temperature variations, and winter conditions frequently expose cultured fish to prolonged cold stress during critical production phases.

To mitigate these effects, nutritional strategies using functional feed additives have gained increasing attention. Plant-derived bioactive compounds, rich in polyphenols and flavonoids, have demonstrated strong antioxidant and immunostimulatory properties, enhancing endogenous defense systems and improving stress tolerance under adverse conditions (8). In this context, *Wolffia globosa* (wolffia), a rootless duckweed of the family Lemnaceae, has emerged as a promising feed resource. It is rich in protein (25–40%), essential amino acids, and bioactive compounds such as gallic acid, chlorogenic acid, kaempferol, and quercetin, which possess potent antioxidant and stress-mitigating properties (9, 10).

Despite these advantages, existing studies have primarily focused on the nutritional value and use of wolffia as fresh biomass in aquaculture (11–14). Information on the functional role of wolffia-derived extracts in modulating antioxidant defense, innate immunity, and immune-related gene expression under low-temperature stress remains limited. Therefore, the present study was conducted to evaluate the effects of dietary supplementation with *Wolffia globosa* extract on immune responses, antioxidant defense, and gene expression in *Labeo rohita* under winter conditions, aiming to provide insights into its potential as a functional dietary intervention for enhancing fish resilience to cold stress.

## 2 Material and methods

### 2.1 Preparation of wolffia extract

Fresh wolffia was collected from a pilot-scale earthen pond at the College of Fisheries, Lembucherra, Tripura. The biomass was thoroughly washed with clean pond water to remove adhering debris, and holdfasts and epiphytes were manually removed. The cleaned biomass was then freeze-dried for 24 h at below −95 °C using a laboratory freeze dryer (ScanVac CoolSafe, LaboGene, Denmark).

The extract was prepared following our previously optimized protocol Yadav et al. (15), in which the drying method and extraction solvent were selected to maximize the recovery of bioactive compounds. Briefly, the freeze-dried wolffia powder was soaked in 100% ethanol at a 1:10 (w/v) ratio and incubated in a cooling incubator shaker (iGene Labserve, India) for 48 h at 4 °C and 120 rpm. The mixture was then centrifuged (Eppendorf 5910 Ri, Germany) at 5000 rpm for 10 min, and the supernatant was filtered through Whatman No. 1 filter paper. The filtrate was concentrated using a rotary evaporator (IKA HB 10 RV 6, Germany) at 40 °C and subsequently freeze-dried.

### 2.2 Formulation and preparation of the experimental diets

Four isonitrogenous (25% crude protein) and isocaloric (16.5 MJ kg^−1^ gross energy) experimental diets were formulated using locally available feed ingredients (**Table 1**). The experimental diets were supplemented with wolffia extract (WGE) at inclusion levels of 0 (control), 0.5, 1.5, and 3.0 g kg^−1^. Accordingly, the diets were designated as C (0 g kg^−1^), WGE_0.5_ (0.5 g kg^−1^), WGE_1.5_ (1.5 g kg^−1^), and WGE_3.0_ (3.0 g kg^−1^), respectively. All ingredients were weighed according to the formulation and thoroughly mixed to obtain a homogeneous blend. An adequate amount of water was added to form a uniform dough, which was then steam-cooked in an autoclave for 20 min and allowed to cool to room temperature. Subsequently, the pre-weighed feed additives and WGE were incorporated and mixed thoroughly. The prepared dough was passed through an automatic pelletizer to produce pellets of 2 mm diameter. The pellets were oven-dried at 60 °C until the moisture content was reduced to below 10%, then packed in labeled airtight bags and stored at room temperature until use. The proximate composition of the experimental diets is presented in **Table 1**.

**Table 1:** Formulation and proximate composition of the experimental diets.

Ingredients composition	^1^Treatments			
(g kg ^-1^)	C	WGE_0.5_	WGE_1.5_	WE_3.0_
^2^FM ^a^	140.00	140.00	140.00	140.00
^3^SBM ^a^	190.00	190.00	190.00	190.00
^4^ MOC ^a^	190.00	190.00	190.00	190.00
^5^ RB ^a^	150.00	150.00	150.00	150.00
Broken wheat ^a^	150.00	150.00	150.00	150.00
Maize flour ^a^	130.00	130.00	130.00	130.00
^6^ CMC ^b^	10.00	10.00	10.00	10.00
Oil ^a^	20.00	20.00	20.00	20.00
*Vit-Min mix	10.00	10.00	10.00	10.00
α-cellulose ^b^	10.00	9.50	8.50	7.00
Wolffia extract ^c^	0.00	0.50	1.50	3.00
Total	1000.00	1000.00	1000.00	1000.00
Proximate composition (g kg^-1^, dry weight basis)				
Crude protein	24.69	24.74	24.59	24.70
Crude fat	5.27	5.07	4.87	4.53
Crude fiber	9.73	11.03	10.07	11.20
^7^NFE	949.10	948.68	950.10	949.31
Total ash	11.21	10.48	10.38	10.26
^8^GE (MJ kg^-1^)	17.08	17.06	17.08	17.05
^9^DE (MJ kg^-1^)	16.50	16.48	16.50	16.47

^1^ C- Control (without wolffia extract); WGE_0.5_-basal feed+0.5 g kg^-1^ wolffia extract; WGE_1.5_- basal feed+1.5 g kg^-1^ wolffia extract; WGE_3.0_- basal feed+3.0 g kg^-1^ wolffia extract.

^2^ FM, Fish Meal.

^3^ SBM, Soyabean Meal.

^4^ MOC, Mustard Oil Cake.

^5^ RB, Rice bran.

^6^ CMC, Carboxy Methyl Cellulose.

^a^ Purchased from local dealers, Agartala, Tripura, India.

^b^ Procured from HiMedia Ltd., India.

^c^ *Wolffia globosa* collected from pilot scale culture pond of the college of Fisheries, Tripura, India.

^7^ NFE (Nitrogen free extract) = [1000 - (crude protein+ crude lipid + crude fiber + total ash)].

^8^ GE, gross energy (Kcal/100 g) = [5.7 × CP % + 9.4 × EE% + 4.1 × NFE%] (Halver, 1976).

^9^ DE, digestible energy (Kcal/100 g) = [4 × CP % + 9 × EE% + 4 × NFE%] (Halver, 1976).

* Source- Agrimin; Composition of vitamin mineral mix (quantity kg^-1^): vitamin A- 55,00,000 IU; vitamin D3- 11,00,000 IU; vitamin B2- 2,000 mg; vitamin E- 750 mg; vitamin K- 1,000 mg; vitamin B6- 1,000 mg; vitamin B12–6 mcg; calcium pantothenate—2,500 mg; nicotinamide—10 g; choline chloride—150 g; Mn—27,000 mg; Cu—2,000 mg; I—1,000 mg; Zn—5,000 mg; Fe—7,500 mg; Co—450 mg; L-lysine—10 g; DL—methionine- 10 g; selenium—125 mg.

### 2.3 Experimental animal, experimental design, and setup

The experiment was conducted in twelve outdoor cement tanks (2 m × 1.2 m × 1 m) with a 6–8 cm soil base at the College of Fisheries, Lembucherra, Tripura, India. Prior to stocking, the tanks were sun-dried, thoroughly cleaned, and disinfected using slaked lime at a rate of 250 kg ha^−1^, after which they were filled with groundwater. Tank fertilization was carried out using raw cow dung at 1.2 kg tank^−1^ (5000 kg ha^−1^) and single superphosphate at 2.4 g tank^−1^ (10 kg ha^−1^). Healthy *L. rohita* fingerlings (average weight: 8.35 ± 0.5 g) were obtained from the college farm and acclimatized for two weeks under outdoor tank conditions. Following the establishment of a healthy plankton population after one week of fertilization, a total of 240 fingerlings were randomly distributed among four dietary treatments in triplicate, with 20 fish stocked per tank, following a completely randomized design. Fish were fed the experimental diets at 4% of body weight per day, divided into two equal rations provided at 10:00 and 17:00 h, for a period of 75 days (24 December 2024 to 8 March 2025). Feed was provided using feeding trays installed in each tank.

Physicochemical water quality parameters, including temperature, dissolved oxygen (DO), pH, and total ammonia, were monitored at fortnightly intervals throughout the experimental period. Temperature and DO were recorded in the morning (08:00–08:30 h) using an EXO multiparameter sonde (YSI Inc., USA), while pH was measured with a digital pH meter (Model 335, Systronics). Total ammonia was analyzed using a continuous flow analyzer (SA3000/5000, Skalar) with an autosampler (SA 1100).

### 2.4 Proximate composition of feed and whole-body carcass

The proximate composition of experimental diets and whole-body carcass was determined following standard procedures of AOAC (16). Moisture content was measured by drying pre-weighed samples in a porcelain crucible at 105 °C for 24 h. Crude protein was analyzed using the Kjeldahl method after digestion with concentrated H_2_SO_4_ and a catalyst mixture (K_2_SO_4_:CuSO_4_, 7:1) at 410 °C for 1 h 45 min (Kel Plus KES 12B E, Pelican Equipments, Chennai, India), followed by distillation using an automated system (Kjeltec 8400, FOSS, Denmark). Crude lipid content was determined using a Soxtec extraction system (ST 243, FOSS, Denmark), and crude fiber was estimated using a Fibertec system (FT 122, FOSS, Denmark).

### 2.5 Blood and tissue sampling

From each tank, six fishes (n =6) were selected randomly, and anaesthetized with clove oil @ 50 μg/L. One ml hypodermal medical syringe (24-gage needle) previously rinsed in a 2.7% EDTA solution was used to draw blood from the caudal peduncle region of the fish. Blood samples were pooled and transferred to the EDTA coated centrifuge tubes, which were then gently shaken to avoid hemolysis. For plasma, blood was drawn into centrifuge tubes (without EDTA) which was immediately centrifuged at 3000 rpm for 10–15 min. The supernatant plasma was then pooled, and stored in the deep freezer at 20 °C, until use. For enzymatic analysis, tissues (gut, liver and muscle) were collected aseptically by dissecting over a cold plate. Using 0.25 M chilled sucrose solution, 5% tissue homogenate was made, and centrifuged at 5000 rpm (15 min) and then stored at 20 °C.

### 2.6 Non-specific immune responses

#### 2.6.1 Serum anti-protease activity

Serum anti-protease activity was assessed according to the method of Sahoo et al. (17) with minor modifications. Briefly, 10 µL of serum was mixed with 100 µL of trypsin (Type I, bovine pancreas; Sigma, USA) prepared in Tris–HCl buffer (50 mM, pH 8.2). Control blanks consisted of 110 µL phosphate-buffered saline (PBS), while reference samples were prepared by combining 10 µL PBS with 100 µL trypsin. The mixtures were incubated at 25 °C for 30 min, after which 1 mL of 0.2% casein solution (HiMedia, India) prepared in PBS was added and incubation continued for an additional 15 min. Enzymatic activity was terminated by the addition of 500 µL of 10% trichloroacetic acid (TCA). Samples were then centrifuged at 10,000 × g for 15 min at 25 °C, and the absorbance of the resulting supernatant was measured at 280 nm. Anti-protease activity was expressed as percentage inhibition using the following formula:


$$\text{Percent inhibition}=\frac{\text{OD of reference}-\text{OD of sample}\;}{\text{OD of reference}}\times 100$$

#### 2.6.2 Serum myeloperoxidase activity

Serum myeloperoxidase (MPO) activity was estimated following the method described by Sahoo et al. (17) with slight modifications. Briefly, 10 µL of serum was diluted with 90 µL of Hank’s balanced salt solution (HBSS) without Ca^2+^ or Mg^2+^ in a 96-well microplate. Subsequently, 35 µL of a freshly prepared reaction mixture containing 20 mM 3,3′,5,5′-tetramethylbenzidine hydrochloride (TMB; Genei, India) and 5 mM hydrogen peroxide (1:20 dilution) was added to each well. The reaction was allowed to proceed for 2 min at room temperature and was then terminated by the addition of 35 µL of 4 M sulfuric acid. The resulting color intensity was measured as optical density at 450 nm using a microplate reader.

#### 2.6.3 Respiratory oxidative burst activity

The respiratory oxidative burst activity of neutrophils was assessed by measuring the reduction of nitroblue tetrazolium (NBT) to formazan, following the method of Sahoo et al. (17) with minor modifications. Briefly, heparinized blood (0.1 mL) was mixed with an equal volume of 0.2% NBT solution (Sigma, USA) in a 2 mL microcentrifuge tube and incubated at 30 °C for 30 min. After incubation, 50 µL of the reaction mixture was transferred to 1.0 mL of N, N-dimethylformamide (DMF; Qualigens, India) in a glass tube and centrifuged at 3,000 × g for 5 min at 30 °C. The absorbance of the supernatant, representing the extent of NBT reduction, was measured at 540 nm using DMF as the blank.

#### 2.6.4 Lysozyme activity

Lysozyme activity was measured following the method of Anderson and Siwicki (18). Briefly, 0.1 mL of serum was added to 2 mL microcentrifuge tubes, then 0.9 mL of a *Micrococcus lysodeikticus* suspension (0.75 mg mL^−1^ in PBS, pH 6.2; Sigma, St. Louis, MO, USA) was added. Immediately after bacterial addition, absorbance at 450 nm was recorded at 1-min intervals for 10 min using a spectrophotometer. Lysozyme activity was calculated from the mean change in absorbance per minute and quantified against a standard curve prepared with hen egg-white lysozyme (Sigma-Aldrich).

### 2.7 Serum biochemical parameters

Total plasma protein was quantified using a commercial diagnostic kit (Coral Clinical Systems, India) based on the Biuret reaction. Plasma albumin concentration was determined using an albumin diagnostic kit (Coral Clinical Systems, India) employing the bromocresol green dye-binding method (19). Globulin concentration was calculated as the difference between total plasma protein and albumin, and the albumin-to-globulin (A/G) ratio was obtained by dividing albumin by globulin.

Serum glucose concentration was estimated using a commercial glucose diagnostic kit (Coral Clinical Systems, India) based on the glucose oxidase–peroxidase (GOD–POD) method described by Trinder (20). In this assay, glucose is enzymatically oxidized to gluconic acid with the concomitant production of hydrogen peroxide, which participates in a colorimetric reaction for glucose determination. Serum glutamic oxaloacetic transaminase (SGOT/AST), serum glutamic pyruvic transaminase (SGPT/ALT), alkaline phosphatase (ALP), direct bilirubin, triglycerides, creatine kinase MB fraction (CK-MB), and electrolytes such as sodium (Na^+^) and potassium (K^+^), were analyzed using commercially available enzymatic assay kits (Coral Clinical Systems, India) following the manufacturer’s instructions.

### 2.8 Oxidative stress biomarkers

Oxidative stress biomarkers, including superoxide dismutase (SOD), catalase (CAT), and malondialdehyde (MDA), were assessed in serum and liver tissues. CAT activity was measured following Takahara et al. (21) by initiating the reaction with the addition of 1 mL hydrogen peroxide (H_2_O_2_). SOD activity was determined according to Misra and Fridovich (22), where the reaction was initiated by adding 0.4 mL epinephrine, and the change in absorbance was recorded at 480 nm for 2 min using a UV–visible spectrophotometer. One unit of SOD activity was defined as the amount of protein required to inhibit epinephrine auto-oxidation by 50%. Malondialdehyde levels were quantified using a commercial MDA assay kit based on the thiobarbituric acid (TBA) method, with absorbance measured at 532 nm.

### 2.9 RNA extraction and cDNA synthesis

Total RNA was extracted from liver, kidney and muscle tissues of *L. rohita* using TRIzol reagent (Invitrogen, USA) following the manufacturer’s instructions. RNA concentration and purity were assessed using a NanoDrop spectrophotometer (Thermo Scientific, USA), and RNA integrity was verified by 1% agarose gel electrophoresis. High-quality RNA was reverse-transcribed into first-strand complementary DNA (cDNA) using a commercial cDNA synthesis kit (Fermentas, USA) according to the manufacturer’s protocol.

### 2.10 Quantitative real-time PCR analysis

Quantitative real-time PCR (qPCR) was performed to evaluate the expression of selected stress- and immune-related genes, including heat shock protein 70 (*hsp70*), heat shock protein 90 (*hsp90*), inducible nitric oxide synthase (*inos*), complement component 3 (*c3*), tumor necrosis factor-alpha (*tnf-α*), interferon-gamma (*ifn-γ*), and superoxide dismutase (*sod*). Primer sequences used in this study are listed in **Table 2**. qPCR amplification was carried out on a QuantStudio™ 5 Real-Time PCR System (Applied Biosystems, USA) using SYBR Green chemistry. Each 10 μL reaction contained 5 μL SYBR Green PCR Master Mix, 1.5 μL each of forward and reverse primers, 1 μL of cDNA template, and 2.5 μL of nuclease-free water. All reactions were performed in triplicate to ensure technical reproducibility. The housekeeping gene *β-actin* was used as an internal control for normalization of gene expression. The thermal cycling conditions consisted of an initial denaturation at 95 °C for 10 min, followed by 40 cycles of denaturation at 95 °C for 15 s and annealing/extension at 60 °C for 1 min. Amplification specificity was confirmed by melt-curve analysis. Relative gene expression levels were calculated using the 2^−^ΔΔCt method (23).

**Table 2:** *Labeo rohita* specific gene primer sequences used for the quantitative real-time PCR.

Name of the Gene	Primer sequences (5 ′ to 3 ′) (F: Forward, R: Reverse)	Annealing temp (°C)	Efficiency (%)	GenBank accession number
*ß-actin*	**F:** GACTTCGAGCAGGAGATGG **R:** CAAGAAGGATGGCTGGAACA	53	99.8	XM035292064
*hsp-70*	**F:** CTGTACGAGGGCATCGACTT **R:** GTCCATCTTGGCGTCTCTCA	58	98.6	KM369886
*hsp-90*	**F:** GGAAATCTTCCTCCGAGAGC **R:** CCGAATTGACCGATCATAGA	54	96.8	EU306564
*inos*	**F:** GGAGGTACGTCTGCGAGGAGGCT **R:** CCAGCGCTGCAAACCTATCATCCA	59	97.5	AJ242906
*sod*	**F:** CATGGTGAAGAAGGCTGTT **R:** TCATCAGTGGGCTAAGTGC	56	99.2	MN190715
*c3*	**F:** CCCTGGACAGCATTATCACTCA **R:** GCCTTCACAAGTGCCAACAC	58	95.4	AM773825
*tnf-α*	**F:** AGGCGGCTTGAAAGTAGTGG **R:** TATGCAGAACGTCGTGGTCC	56	97.9	FN543477
*ifn-γ*	**F:** TGGGCGATAAAGGCTGATGATC **R:** ACGCGCTTCAGCTCGAA	50	96.1	HQ667144

F; Forward and R; Reverse.

### 2.11 Statistical analysis

Data were analyzed using the IBM SPSS, version 27.0 (IBM Corp., Armonk, NY, USA). One-way analysis of variance (ANOVA) was employed to assess differences among treatment means, followed by Duncan’s Multiple Range Test (DMRT) for pairwise comparisons at *p* < 0.05. All results are presented as mean ± standard error (SE). Graphs were generated using GraphPad Prism (10.6.1).

## 3 Results

### 3.1 Physico-chemical parameters of water

Temporal variations in water quality parameters during the experimental period are presented in **Table 3**. Water temperature ranged from 18.04 to 22.54 °C, while morning DO levels varied between 6.35 and 7.84 mg L^−1^. The pH remained slightly alkaline, ranging from 7.63 to 8.23. Total ammonia concentrations across all experimental tanks were low, ranging from 0.08 to 0.23 mg L^−1^.

**Table 3:** Temporal variation of physico-chemical water quality parameters during the 75-days experimental period.

Days	Treatment	Temperature (°C)	DO (mg L^−1^)	pH	Total ammonia (mg L^−1^)
0	C	19.38 ± 0.18	6.60 ± 0.03	7.83 ± 0.02	0.13 ± 0.01
	WGE_0.5_	19.90 ± 0.11	6.57 ± 0.05	7.83 ± 0.03	0.13 ± 0.03
	WGE_1.5_	19.64 ± 0.14	6.60 ± 0.12	7.77 ± 0.04	0.12 ± 0.02
	WGE_3.0_	19.41 ± 0.13	6.95 ± 0.02	7.84 ± 0.04	0.14 ± 0.04
15	C	18.48 ± 0.04	7.61 ± 0.08	7.80 ± 0.03	0.14 ± 0.01
	WGE_0.5_	18.27 ± 0.10	7.69 ± 0.04	7.79 ± 0.02	0.16 ± 0.03
	WGE_1.5_	18.28 ± 0.04	7.54 ± 0.10	7.81 ± 0.02	0.16 ± 0.01
	WGE_3.0_	18.36 ± 0.29	7.45 ± 0.07	7.73 ± 0.02	0.15 ± 0.03
30	C	18.67 ± 0.07	7.54 ± 0.23	7.70 ± 0.01	0.16 ± 0.01
	WGE_0.5_	18.31 ± 0.07	7.76 ± 0.05	7.68 ± 0.03	0.18 ± 0.01
	WGE_1.5_	18.45 ± 0.04	7.55 ± 0.07	7.71 ± 0.01	0.20 ± 0.01
	WGE_3.0_	18.16 ± 0.03	7.60 ± 0.11	7.67 ± 0.02	0.19 ± 0.01
45	C	19.67 ± 0.07	6.66 ± 0.06	7.84 ± 0.01	0.17 ± 0.01
	WGE_0.5_	19.31 ± 0.07	6.69 ± 0.05	7.80 ± 0.02	0.19 ± 0.01
	WGE_1.5_	19.45 ± 0.04	6.76 ± 0.08	7.83 ± 0.01	0.18 ± 0.00
	WGE_3.0_	19.16 ± 0.03	6.74 ± 0.09	7.83 ± 0.01	0.20 ± 0.01
60	C	20.91 ± 0.06	6.70 ± 0.01	8.02 ± 0.11	0.16 ± 0.01
	WGE_0.5_	20.84 ± 0.05	6.66 ± 0.03	8.20 ± 0.02	0.18 ± 0.02
	WGE_1.5_	20.85 ± 0.07	6.78 ± 0.03	8.08 ± 0.02	0.17 ± 0.01
	WGE_3.0_	20.91 ± 0.07	6.75 ± 0.03	8.11 ± 0.04	0.20 ± 0.01
75	C	22.03 ± 0.25	6.49 ± 0.07	8.09 ± 0.06	0.17 ± 0.01
	WGE_0.5_	22.15 ± 0.10	6.56 ± 0.11	8.09 ± 0.03	0.16 ± 0.03
	WGE_1.5_	21.90 ± 0.06	6.53 ± 0.05	8.12 ± 0.03	0.21 ± 0.01
	WGE_3.0_	21.93 ± 0.07	6.59 ± 0.04	8.12 ± 0.02	0.20 ± 0.01

Values are expressed as mean ± standard error (n = 3). C- Control (without wolffia extract); WGE_0.5_-basal feed+0.5 g kg^-1^ wolffia extract; WGE_1.5_- basal feed+1.5 g kg^-1^ wolffia extract; WGE_3.0_- basal feed+3.0 g kg^-1^ wolffia extract.

### 3.2 Whole-body proximate composition

As presented in **Table 4**, dietary inclusion of WGE altered the carcass composition of *L. rohita* fingerlings reared during the winter season (*p* < 0.05). Crude protein content exhibited a dose-dependent increase, reaching the highest value at 3.0 g kg^−1^ WGE (18.01%), followed by 1.5 g kg^−1^ (16.21%), compared to the control (15.32%). A similar trend was observed for crude lipid content, with the maximum level recorded at 3.0 g kg^−1^ WGE (4.63%). Ash content also increased in WGE-fed groups, with higher values at 3.0 g kg^−1^ (2.83%) and 1.5 g kg^−1^ (2.82%) relative to the control. In contrast, moisture content remained unchanged across treatments (*p* > 0.05).

**Table 4:** Whole body composition of *L. rohita* fed different experimental diets after 75 days of rearing (% wet weight basis).

Proximate Composition (%)	C	WGE_0.5_	WGE_1.5_	WGE_3.0_	*p* value
Moisture (%)	74.43 ± 0.14^a^	74.76 ± 0.04^a^	74.37 ± 0.30^a^	74.91 ± 0.26^a^	0.291
Protein (%)	15.32 ± 0.38^c^	15.80 ± 0.29^c^	16.21 ± 0.20^ab^	18.01 ± 1.06^a^	0.000
Fat (%)	4.44 ± 0.03^b^	4.02 ± 0.05^c^	4.16 ± 0.04^c^	4.63 ± 0.05^a^	0.000
Ash (%)	2.67 ± 0.03^b^	2.82 ± 0.01^a^	2.65 ± 0.01^b^	2.83 ± 0.02^a^	0.053

Data expressed as Mean ± SE (n = 3); Mean values in the same row with different superscript differ significantly (P< 0.05). C- Control (without wolffia extract); WGE_0.5_-basal feed+0.5 g kg^-1^ wolffia extract; WGE_1.5_- basal feed+1.5 g kg^-1^ wolffia extract; WGE_3.0_- basal feed+3.0 g kg^-1^ wolffia extract.

### 3.3 Non-specific immune responses

#### 3.3.1 Anti-protease activity

**Figure 1:**
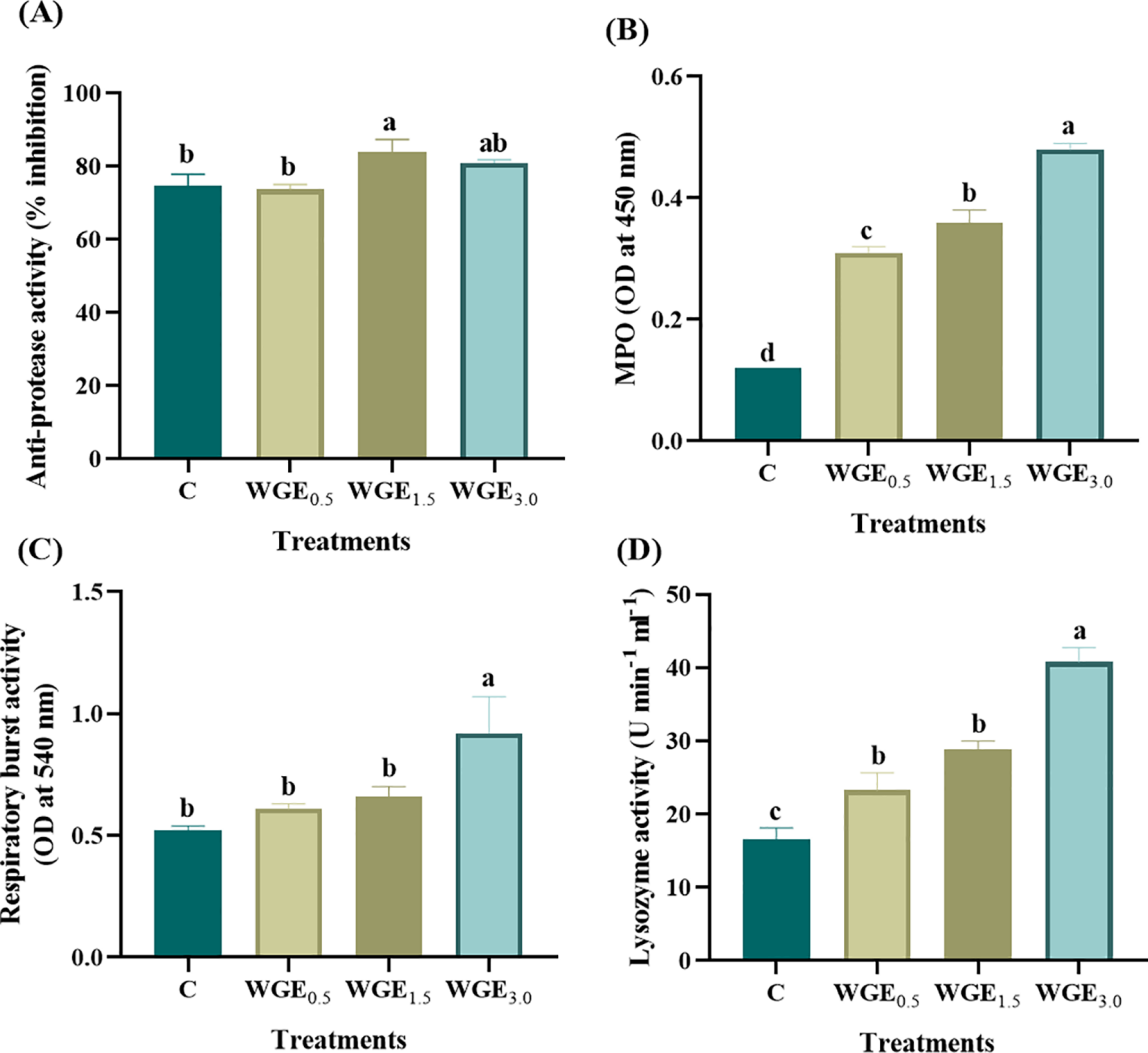
Effects of dietary WGE supplementation on non-specific immune responses in *L. rohita*. **(A)** Anti-protease activity (APA), **(B)** Myeloperoxidase (MPO), **(C)** Respiratory burst activity, **(D)** Lysozyme activity. All values are expressed as mean ± SE (n =3). Bars with different letters indicate significant differences among treatments (*p* < 0.05). C- Control (without wolffia extract); WGE_0.5_-basal feed+0.5 g kg^-1^ wolffia extract; WGE_1.5_- basal feed+1.5 g kg^-1^ wolffia extract; WGE_3.0_- basal feed+3.0 g kg^-1^ wolffia extract.

Serum anti-protease activity (APA) in *L. rohita* was significantly enhanced (*p* < 0.05) following dietary supplementation with WGE (**Figure 1A**). Fish fed 1.5 and 3.0 g kg^−1^ WGE exhibited increases of 12.32% and 8.22%, respectively, compared with the control group, indicating improved innate immune responsiveness under winter rearing conditions.

#### 3.3.2 Myeloperoxidase activity

Serum myeloperoxidase (MPO) activity in *L. rohita* exhibited a clear dose-dependent response to dietary WGE supplementation. A significant elevation (*p* < 0.05) was observed at the highest inclusion level, with the 3.0 g kg^−1^ group showing the greatest activity, followed by the 1.5 g kg^−1^ treatment (**Figure 1B**). MPO activity increased by approximately 300% and 200% over the control in the 3.0 and 1.5 g kg^−1^ groups, respectively, indicating a marked enhancement of innate immune defense.

#### 3.3.3 Respiratory oxidative burst activity

Serum respiratory burst activity of phagocytes, assessed via the NBT assay, was significantly elevated (*p* < 0.05) in fish receiving 3.0 g kg^−1^ WGE compared with all other treatments, representing a 76.92% increase over the control (**Figure 1C**). No significant differences were observed between the lower WGE inclusion levels and the control group.

#### 3.3.4 Lysozyme activity

Lysozyme activity in serum was significantly higher (*p* < 0.05) in fish fed the 3.0 g kg^−1^ WGE diet, showing increases of 41.56%, 74.95%, and 146.94% compared to the 1.5 g kg^−1^, 0.5 g kg^−1^ WGE diet, and the control group, respectively (**Figure 1D**).

### 3.4 Serum biochemical parameters

**Figure 2:**
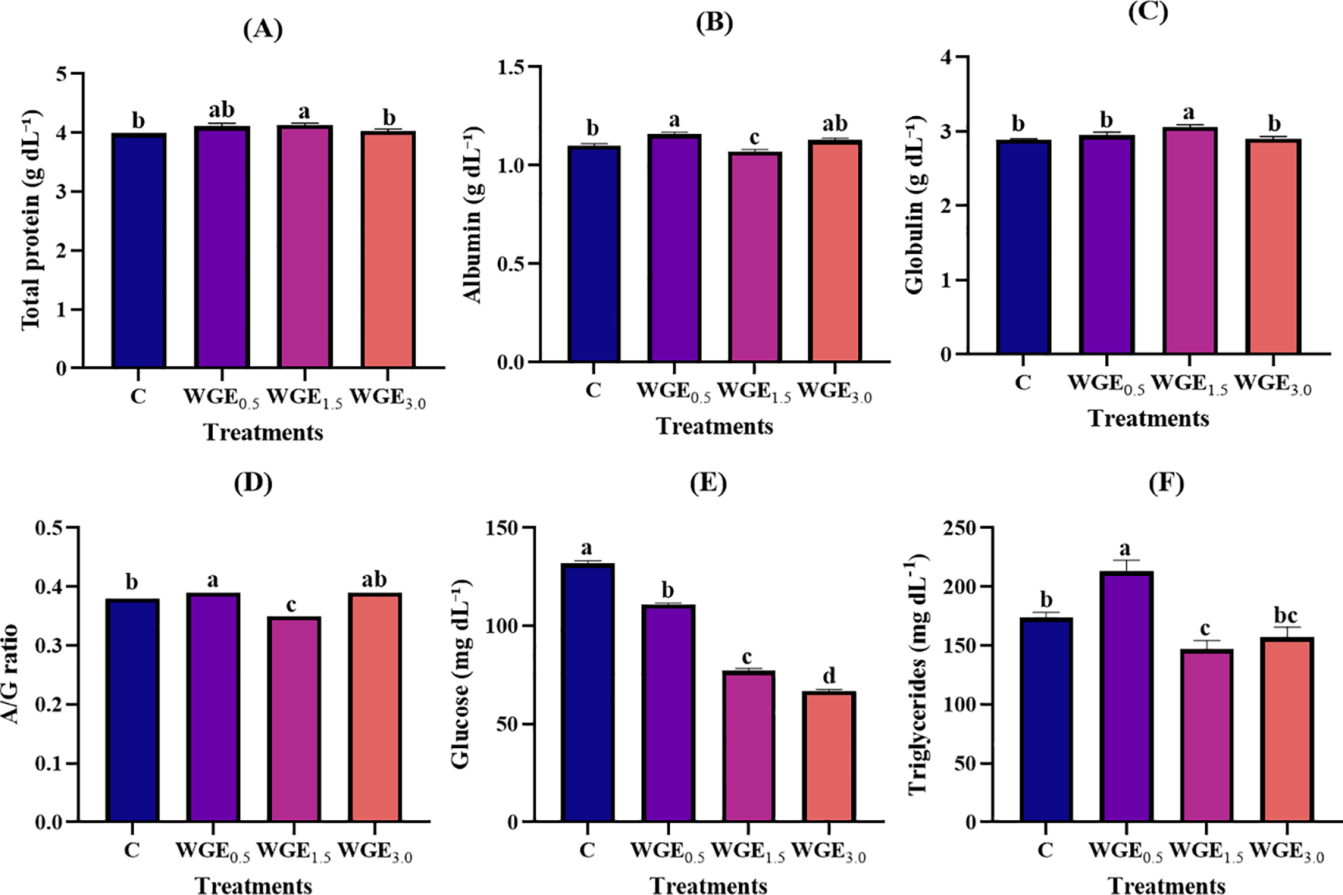
Effect of dietary WGE supplementation on serum biochemical indices in *L. rohita*. **(A)** Total protein, **(B)** albumin, **(C)** globulin, **(D)** albumin-to-globulin (A/G) ratio, **(E)** glucose, and **(F)** triglycerides. All values are expressed as mean ± SE (n =3). Bars with different letters indicate significant differences among treatments (*p* < 0.05). C- Control (without wolffia extract); WGE_0.5_-basal feed+0.5 g kg^-1^ wolffia extract; WGE_1.5_- basal feed+1.5 g kg^-1^ wolffia extract; WGE_3.0_- basal feed+3.0 g kg^-1^ wolffia extract.

**Figure 3:**
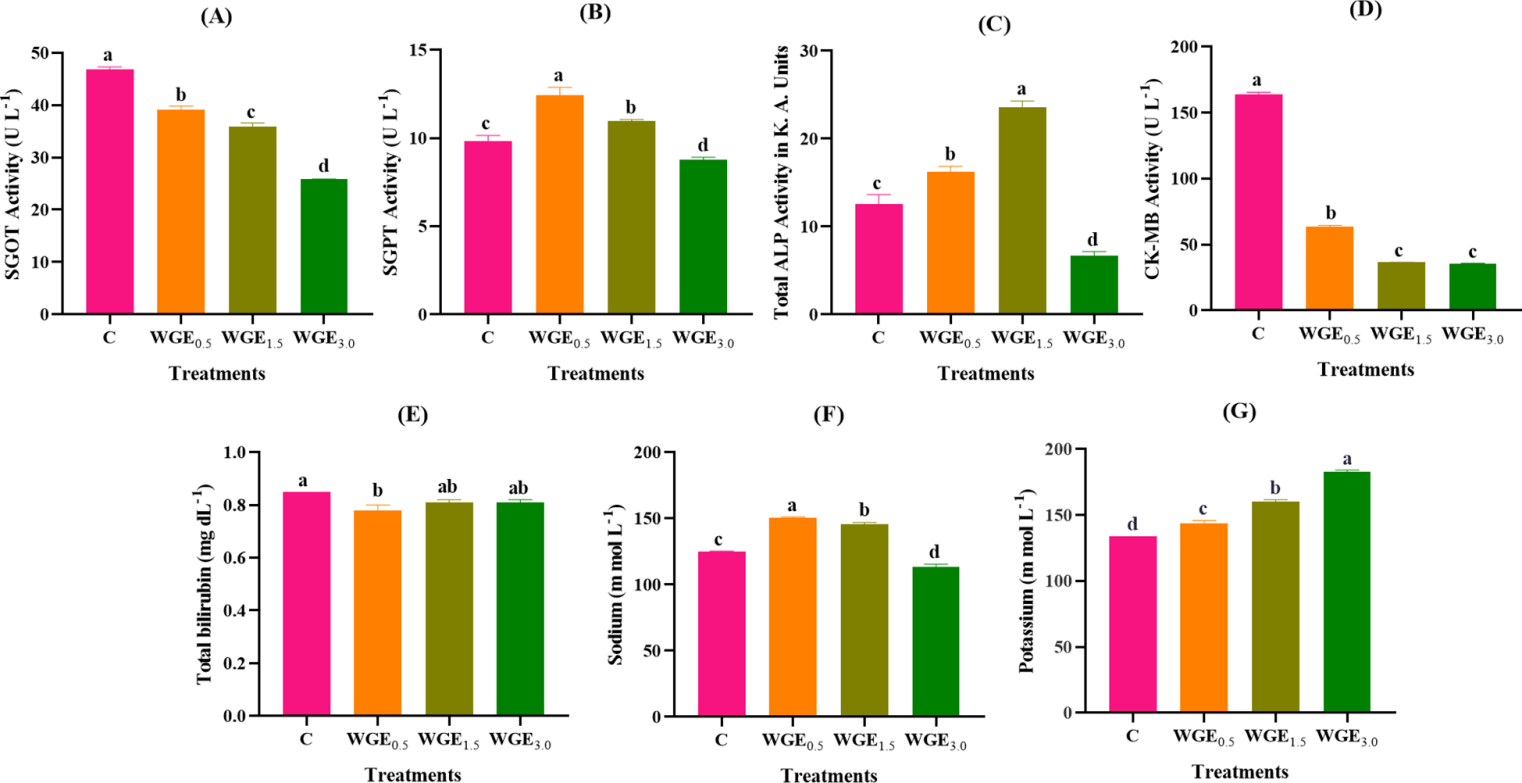
Effect of dietary WGE supplementation on serum enzyme activities and electrolyte profiles in *L. rohita*. **(A)** SGOT, **(B)** SGPT, **(C)** alkaline phosphatase (ALP), **(D)** creatine kinase-MB (CK-MB), **(E)** total bilirubin, **(F)** sodium (Na^+^), and **(G)** potassium (K^+^). All values are expressed as mean ± SE (n =3). Bars with different letters indicate significant differences among treatments (*p* < 0.05). C- Control (without wolffia extract); WGE_0.5_-basal feed+0.5 g kg^-1^ wolffia extract; WGE_1.5_- basal feed+1.5 g kg^-1^ wolffia extract; WGE_3.0_- basal feed+3.0 g kg^-1^ wolffia extract.

The serum biochemical parameters, including total protein, albumin, globulin, A/G ratio, glucose, triglycerides, SGOT, SGPT, ALP, CK-MB, total bilirubin, Na^+^, and K^+^, are presented in **Figure 2** and **Figure 3**. Total protein levels were elevated in fish fed 1.5 and 0.5 g kg^−1^ WGE compared to the control (**Figure 2A**). Albumin showed a similar response, with higher values at 0.5 and 3.0 g kg^−1^ WGE, whereas the lowest level was recorded at 1.5 g kg^−1^ (**Figure 2B**). In contrast, globulin content peaked at 1.5 g kg^−1^ WGE, while remaining comparable among the other treatments (**Figure 2C**). Consequently, the A/G ratio followed the pattern of albumin, with higher values at 0.5 and 3.0 g kg^−1^ and a minimum at 1.5 g kg^−1^ WGE (**Figure 2D**).

Serum glucose exhibited a clear declining trend with increasing WGE inclusion, with reductions of 15.78%, 41.52%, and 49.26% at 0.5, 1.5, and 3.0 g kg^−1^, respectively (**Figure 2E**). Triglyceride levels displayed a biphasic response, increasing at 0.5 g kg^−1^ WGE (22.68% above control) but decreasing at higher inclusion levels, with the lowest value observed at 1.5 g kg^−1^ (**Figure 2F**).

Transaminase activities showed distinct patterns in response to WGE supplementation. SGOT decreased progressively with increasing inclusion levels, indicating a dose-dependent reduction (**Figure 3A**). In contrast, SGPT initially increased at lower inclusion levels (0.5 and 1.5 g kg^−1^) before declining at 3.0 g kg^−1^ (**Figure 3B**). A similar biphasic pattern was observed for ALP activity, which increased at 0.5 g kg^−1^ but declined at higher inclusion levels (**Figure 3C**).

CK-MB activity showed a marked and consistent decline with increasing WGE supplementation, indicating a strong dose-dependent response (**Figure 3D**). Likewise, total bilirubin levels decreased across all treatment groups compared to the control (**Figure 3E**).

Serum electrolyte profiles were also modulated by WGE supplementation. Na^+^ levels increased at 0.5 and 1.5 g kg^−1^ but declined at the highest inclusion level (**Figure 3F**). In contrast, K^+^ exhibited a steady dose-dependent increase across all treatments (**Figure 3G**).

### 3.5 Oxidative stress biomarkers

**Figure 4:**
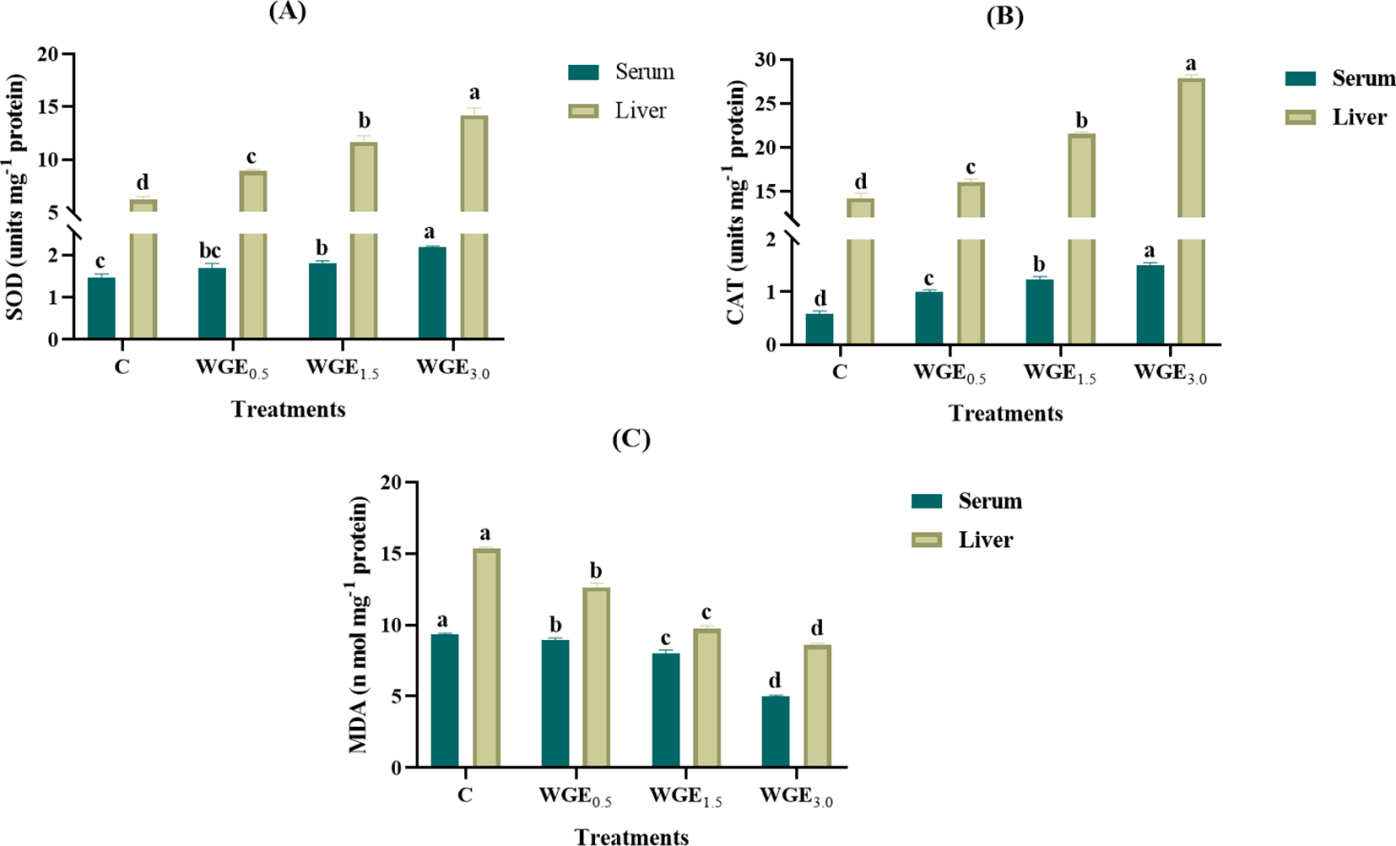
Effects of dietary WGE supplementation on oxidative stress biomarkers in serum and liver tissues of *L. rohita*. **(A)** Superoxide dismutase (SOD), **(B)** catalase (CAT), and **(C)** malondialdehyde (MDA). All values are expressed as mean ± SE (n =3). Bars with different letters indicate significant differences among treatments (*p* < 0.05). C- Control (without wolffia extract); WGE_0.5_-basal feed+0.5 g kg^-1^ wolffia extract; WGE_1.5_- basal feed+1.5 g kg^-1^ wolffia extract; WGE_3.0_- basal feed+3.0 g kg^-1^ wolffia extract.

The activities of oxidative stress biomarkers, including SOD, CAT, and MDA, in serum and liver are presented in **Figure 4**. Overall, dietary WGE supplementation markedly modulated antioxidant status in both tissues (P< 0.05). SOD activity exhibited a clear dose-dependent increase in both liver and serum (**Figure 4A**). In liver, SOD activity rose by 43.25%, 87.30%, and 127.97% at 0.5, 1.5, and 3.0 g kg^−1^ WGE, respectively, compared to the control. A similar but less pronounced trend was observed in serum, with corresponding increases of 15.75%, 23.29%, and 50.00%.

CAT activity followed a comparable pattern, showing progressive enhancement with increasing WGE inclusion (**Figure 4B**). In liver tissue, CAT increased by 13.08%, 51.34%, and 96.27%, while serum CAT exhibited a more pronounced rise of 69.49%, 110.17%, and 154.24% across the respective treatments. In contrast, MDA levels displayed an opposite trend, declining consistently with increasing WGE supplementation (**Figure 4C**). Liver MDA decreased by 17.61%, 36.46%, and 43.97%, while serum levels were reduced by 4.49%, 14.85%, and 46.69% at 0.5, 1.5, and 3.0 g kg^−1^ WGE, respectively, with the lowest values recorded at the highest inclusion level.

### 3.6 Stress and immune gene expression

**Figure 5:**
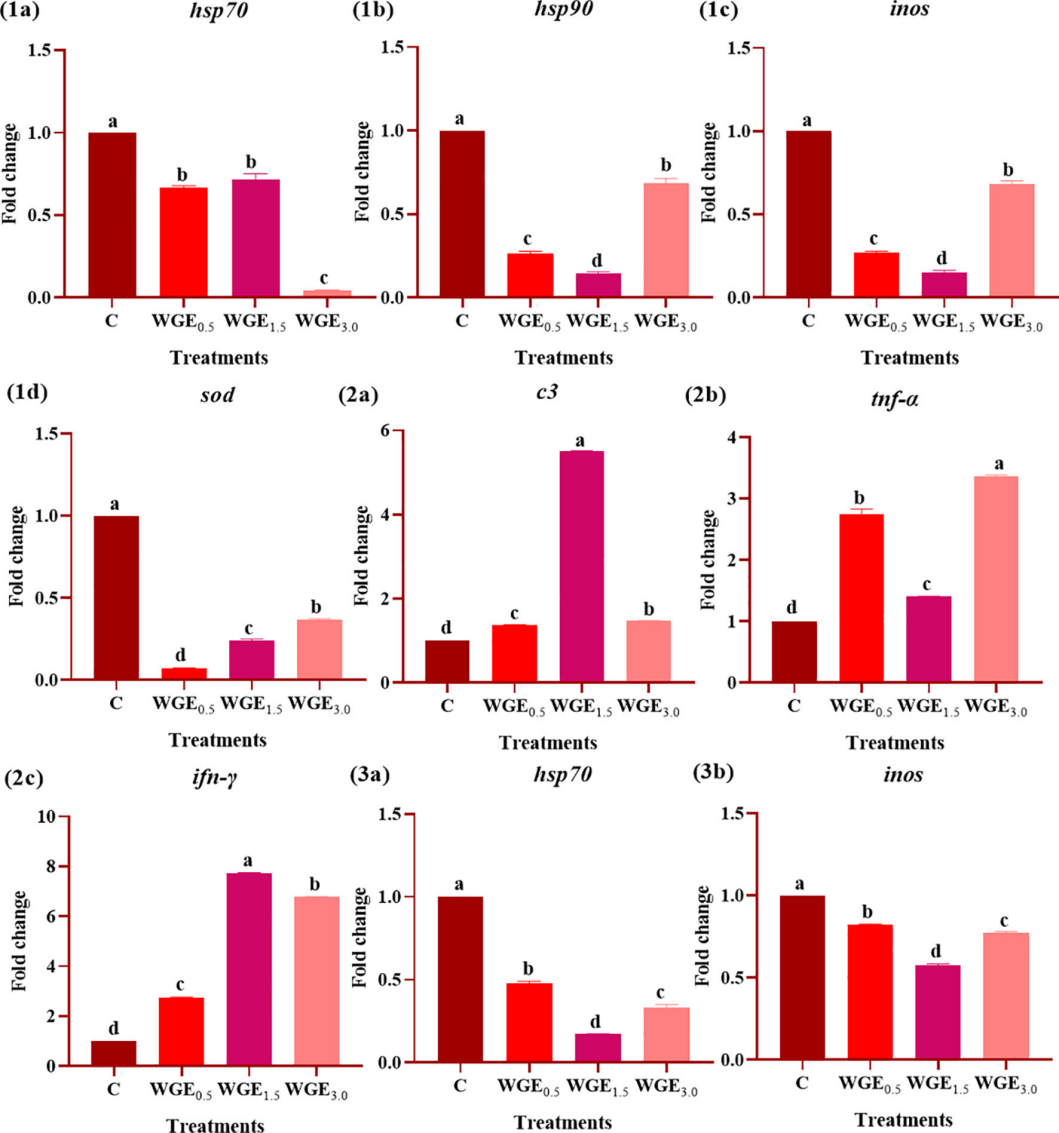
Effect of dietary WGE supplementation on fold change in stress- and immune-related gene expression in liver, kidney, and muscle tissues of fish. **(1A–D)** liver, **(2A-C)** kidney, and **(3A-B)** muscle gene expression. All values are expressed as mean ± SE (n =3). Bars with different letters indicate significant differences among treatments (*p* < 0.05). C- Control (without wolffia extract); WGE_0.5_-basal feed+0.5 g kg^-1^ wolffia extract; WGE_1.5_- basal feed+1.5 g kg^-1^ wolffia extract; WGE_3.0_- basal feed+3.0 g kg^-1^ wolffia extract.

The relative expression of stress- and immune-related genes in liver, kidney, and muscle tissues is presented in **Figure 5**. Overall, dietary WGE supplementation markedly modulated gene expression across all tissues (P< 0.05). In liver tissue, stress-related genes generally exhibited downregulation in response to WGE supplementation. The expression of *hsp70* declined across all treatments, with fold changes of 0.669, 0.714, and 0.042 at 0.5, 1.5, and 3.0 g kg^−1^ WGE, respectively, indicating a pronounced reduction at the highest inclusion level (**Figures 5**, **1A**). A similar pattern was observed for *hsp90* and *inos*, both of which were strongly suppressed at lower inclusion levels (0.5 and 1.5 g kg^−1^), followed by partial recovery at 3.0 g kg^−1^, although expression remained below control levels (**Figures 5**, **1B–C**). In contrast, *sod* expression showed an initial sharp decline at 0.5 g kg^−1^, followed by gradual upregulation with increasing WGE levels, yet remaining lower than the control (**Figures 5**, **1D**).

In kidney tissue, immune-related genes were markedly upregulated with WGE supplementation. The expression of *c3* increased across treatments, with the highest induction at 1.5 g kg^−1^ (5.51-fold) (**Figures 5**, **2A**). Similarly, *tnf-α* expression was elevated in all groups, reaching a maximum at 3.0 g kg^−1^ (3.37-fold) (**Figures 5**, **2B**). The strongest response was observed for *ifn-γ*, which showed substantial upregulation, particularly at 1.5 g kg^−1^ (7.73-fold), followed by slightly lower expression at 3.0 g kg^−1^ (**Figures 5**, **2C**).

In muscle tissue, stress-related gene expression was consistently suppressed by WGE supplementation. The expression of *hsp70* decreased across all treatments, with the greatest reduction at 1.5 g kg^−1^ (0.17-fold) (**Figures 5**, **3A**). A similar downregulation pattern was observed for *inos*, which remained lower than the control across all inclusion levels (**Figures 5**, **3B**).

## 4 Discussion

Temperature is a key environmental factor influencing the physiological, biochemical, and molecular responses of fish, and deviations from the optimal range, particularly during winter, can disrupt metabolic homeostasis, suppress immune function, and impair antioxidant defense systems (24–27). Cold stress further exacerbates these effects by increasing ROS production, leading to oxidative damage, lipid peroxidation, and alterations in antioxidant enzyme activities (28, 29). In this context, dietary interventions using bioactive-rich feed additives have emerged as an effective strategy to enhance innate immunity and improve stress resilience in fish. Duckweeds, particularly *Wolffia globosa*, are rich in nutrients and bioactive compounds and have long been utilized as functional feed resources (9, 10). Given the predominance of innate immune mechanisms in fish (30), strengthening non-specific defenses is critical under stress conditions. Consistent with this, the present study demonstrates that dietary WGE improves immune responses, biochemical status, antioxidant capacity, and gene expression, highlighting its protective role against cold-induced stress.

In the current study, dietary WGE supplementation improved carcass composition of *L. rohita*, as reflected by increased crude protein, lipid, and ash content. Cold stress is known to impair digestion, nutrient absorption, and metabolic efficiency, resulting in poor nutrient utilization and reduced growth performance (31). The higher protein deposition observed in WGE-fed groups may be associated with improved nutrient assimilation and protein retention, potentially mediated by the bioactive compounds present in wolffia, such as phenolics and flavonoids, which can enhance digestive efficiency, reduce oxidative damage, and spare dietary protein for tissue accretion rather than stress adaptation (32). Similarly, the increased lipid content at higher WGE inclusion levels may represent an adaptive metabolic response under low-temperature conditions, where fish accumulate energy reserves to compensate for reduced metabolic activity and feeding behavior (33). In addition, bioactive compounds in WGE may influence lipid metabolism by improving energy utilization and reducing stress-induced catabolism. The elevated ash content further indicates enhanced mineral retention and metabolic efficiency, possibly due to improved nutrient digestibility and absorption, which are essential for maintaining physiological homeostasis during thermal stress (34). Overall, these findings suggest that WGE supplementation supports nutrient partitioning and metabolic adaptation in *L. rohita* under winter stress conditions.

Non-specific immune parameters constitute the primary defense system in fishes and play a vital role in protection against invading pathogens. Immune components such as respiratory burst activity, MPO, and lysozyme function as important humoral and cellular mediators of innate immunity, providing rapid antimicrobial defense (35). In the present study, dietary WGE supplementation markedly enhanced APA, MPO, respiratory burst activity, and lysozyme activity in *Labeo rohita* under winter conditions, indicating improved innate immune competence. Since cold stress is known to suppress immune function and impair primary defense mechanisms in fish (28, 36–38), the observed enhancement suggests that WGE effectively counteracted stress-induced immunosuppression. Mechanistically, the immunostimulatory effects of WGE may be attributed to its rich content of phenolics, flavonoids, and other bioactive compounds, which can modulate immune signaling pathways, stabilize cellular functions, and enhance leukocyte activity (39). The increased APA observed in the present study indicates improved protease inhibition and immune regulation, thereby strengthening host defense against pathogen-derived proteolytic enzymes and stress-associated tissue damage. Similar improvements in APA have been reported in fish supplemented with plant-derived immunostimulants (40–42). The elevated MPO activity, particularly at higher WGE inclusion levels, suggests enhanced neutrophil activation and microbicidal potential, as MPO catalyzes the formation of reactive intermediates involved in pathogen destruction (43). Likewise, the significant enhancement in respiratory burst activity reflects increased phagocytic efficiency and ROS-mediated microbial killing, which are critical components of innate immune defense and are commonly stimulated by phytogenic feed additives (42, 44). Furthermore, the pronounced increase in lysozyme activity indicates strengthened antibacterial defense through enhanced lysis of bacterial cell walls, supporting its role as a reliable indicator of improved innate immune status in fish (45).

Serum biochemical parameters are widely recognized as sensitive indicators of metabolic status, physiological condition, and overall health in fish exposed to environmental stress and dietary interventions (46). In the present study, dietary WGE markedly modulated protein metabolism, glucose homeostasis, lipid utilization, and liver enzyme activities, indicating improved physiological adaptation under winter stress. Cold exposure is known to disrupt metabolic homeostasis by suppressing feeding activity, impairing nutrient assimilation, and increasing energy demand, which collectively alter carbohydrate and protein metabolism (29). The elevated levels of total protein, albumin, and globulin observed in WGE-fed groups suggest enhanced protein synthesis, nutrient utilization, and humoral immune status. Mechanistically, these improvements may be linked to the bioactive compounds present in WGE, particularly phenolics and flavonoids, which can enhance digestive efficiency, reduce oxidative damage, and support hepatic protein synthesis. Similar increases in serum protein fractions have been reported in fish supplemented with plant-derived functional feeds, reflecting improved nutritional and immune status (47).

The marked reduction in serum glucose levels at higher WGE inclusion further indicates improved stress tolerance and metabolic regulation, as stress-induced hyperglycemia is a common secondary response mediated through cortisol-driven glycogenolysis and gluconeogenesis in fish (1, 48). Likewise, modulation of triglyceride levels suggests improved lipid metabolism and energy utilization under low-temperature conditions. In addition, the decline in SGOT, SGPT, and ALP activities indicates improved hepatic integrity and reduced cellular damage, since elevated levels of these enzymes are generally associated with liver dysfunction and metabolic disturbances (49). The substantial reduction in CK-MB activity further suggests alleviation of stress-associated tissue damage and improved physiological stability. These beneficial effects may result from the antioxidant and membrane-stabilizing properties of WGE bioactive compounds, which protect tissues from oxidative injury during cold stress. Comparable improvements in serum biochemical profiles have been reported in *L. rohita* and other fish species fed plant extracts and immunostimulatory feed additives (50–52).

Oxidative stress biomarkers such as SOD, CAT, and MDA are widely recognized indicators of redox balance and cellular integrity in fish. In the present study, dietary WGE enhanced SOD and CAT activities while reducing MDA levels, indicating improved antioxidant defense under winter stress conditions. Low-temperature exposure disrupts metabolic homeostasis and mitochondrial function, resulting in excessive production of ROS and activation of antioxidant enzymes as a compensatory mechanism (6, 53, 54). The enhanced SOD and CAT activities observed in WGE-fed groups may be attributed to the phenolics, flavonoids, and other bioactive compounds present in wolffia, which possess strong free radical scavenging properties and can stimulate endogenous antioxidant systems. Mechanistically, SOD catalyzes the dismutation of superoxide radicals into hydrogen peroxide, which is subsequently detoxified by CAT, thereby limiting ROS accumulation and protecting cellular components from oxidative injury. Similar improvements in antioxidant enzyme activities have been reported in fish supplemented with plant- and algae-derived bioactive extracts, where enhanced antioxidant capacity contributed to improved stress tolerance and physiological stability (38, 40, 44, 55). In contrast, the reduction in MDA levels indicates decreased lipid peroxidation and membrane damage, further confirming the protective role of WGE against oxidative stress. Since MDA is a major end-product of lipid peroxidation and a hallmark of oxidative damage, its decline reflects improved cellular protection, membrane stability, and maintenance of redox homeostasis under cold stress conditions (56).

The expression of stress- and immune-related genes provides valuable insights into the physiological and immunological status of fish under environmental stress conditions (57, 58). In the present study, dietary WGE markedly modulated the expression of stress (*hsp70*, *hsp90*), antioxidant (*sod*), and immune-related genes (*inos*, *c3*, *tnf-α*, and *ifn-γ*), indicating enhanced stress tolerance and immune competence under low-temperature conditions. Cold stress is known to induce the expression of heat shock proteins, particularly *hsp70* and *hsp90*, which function as molecular chaperones involved in protein folding, stabilization, and prevention of stress-induced protein denaturation (29, 59). Therefore, the downregulation of *hsp70* and *hsp90* observed in WGE-fed groups suggests reduced cellular stress and improved maintenance of cellular homeostasis, indicating that dietary supplementation alleviated the stress burden imposed by winter conditions.

Similarly, modulation of *inos* expression reflects altered inflammatory and immune regulation, as *iNOS* is involved in nitric oxide production during immune activation and inflammatory responses (60). The reduced expression of *inos* in liver and muscle tissues suggests attenuation of stress-induced inflammatory processes in WGE-supplemented fish. In contrast, the significant upregulation of *c3*, *tnf-α*, and *ifn-γ* in kidney tissue indicates stimulation of innate immune pathways. Mechanistically, *c3* plays a central role in complement activation and opsonization, *tnf-α* regulates inflammatory signaling and leukocyte activation, while *ifn-γ* enhances macrophage activity and pathogen resistance (61, 62). Similar upregulation of immune-related genes has been reported in fish fed plant-derived immunostimulants, reflecting improved immune responsiveness and disease resistance (63–66).

The modulation of *sod* expression further supports the biochemical findings, indicating coordinated regulation of antioxidant defense at both transcriptional and enzymatic levels. Previous studies have shown that plant-derived bioactive compounds can regulate molecular pathways associated with oxidative stress and immunity, thereby enhancing stress resilience in fish (67–69). The phenolics and flavonoids present in wolffia may therefore contribute to the observed molecular responses through antioxidant and immunomodulatory actions. Collectively, the coordinated regulation of stress, antioxidant, and immune-related genes demonstrates that WGE supplementation mitigates cold-induced stress while enhancing the adaptive and immune capacity of *L. rohita* under winter conditions.

## 5 Conclusion

The present study demonstrates that dietary supplementation with *Wolffia globosa* extract (WGE) effectively mitigates the adverse effects of low-temperature stress in *Labeo rohita*. WGE supplementation enhanced non-specific immune responses, improved serum biochemical profiles, and strengthened antioxidant defense systems, as evidenced by increased SOD and CAT activities and reduced MDA. Moreover, the modulation of stress- and immune-related gene expression, including the downregulation of *hsps* and *inos* and upregulation of key immune genes, indicates improved cellular homeostasis and immune competence. The improvement in carcass composition further suggests enhanced nutrient utilization and metabolic efficiency under winter conditions. Among the tested levels, 1.5 g kg^−1^ WGE appeared to be the most effective dose, providing optimal improvement in immune responses, biochemical balance, and overall physiological status without excessive lipid accumulation, while higher inclusion (3.0 g kg^−1^) showed pronounced antioxidant and immune enhancement but with comparatively less balanced metabolic outcomes. Overall, WGE can be considered a promising functional feed additive for improving stress resilience and health of *L. rohita* under low-temperature conditions.

## Data Availability

The original contributions presented in the study are included in the article/supplementary material. Further inquiries can be directed to the corresponding authors.
